# Association of Chest Compression Depth, Rate, and Spring Resistance with “Quality CardioPulmonary Resuscitation” (QCPR) Scores in a Manikin Model

**DOI:** 10.1007/s10916-026-02469-z

**Published:** 2026-10-05

**Authors:** Špela Metličar, Kasper G Lauridsen, Gregor Štiglic, Nino Fijačko

**Affiliations:** 1 Medical Dispatch Centre Maribor, University Clinical Centre Ljubljana, Ljubljana, Slovenia; 2 Research Center for Emergency Medicine, Department of Clinical Medicine, Aarhus University, Aarhus, Denmark; 3 Department of Medicine, Randers Regional Hospital, Randers, Denmark; 4 Faculty of Health Sciences, University of Maribor, Maribor, Slovenia; 5 Faculty of Electrical Engineering and Computer Science, University of Maribor, Maribor, Slovenia; 6 Usher Institute, University of Edinburgh, Edinburgh, UK; 7 Faculty of Health Sciences, University of Ljubljana, Zdravstvena pot 5, 1000 Ljubljana, Slovenia

**Keywords:** Cardiopulmonary resuscitation, Chest compressions, QCPR, Feedback system, Compression depth, Compression rate, Chest stiffness, Resuscitation training, Manikin study

## Abstract

Training in high-quality chest compressions (CCs) is essential for effective resuscitation; however, it remains unclear how accurately real-time feedback systems, such as Laerdal Medical’s (Stavanger, Norway) Quality CardioPulmonary Resuscitation (QCPR) scoring system, reflect guideline-compliant CC quality. We aimed to investigate how CC depth, rate, and spring resistance affect QCPR scores. We conducted an experimental study using a Resusci Anne QCPR manikin (Laerdal Medical, Stavanger, Norway) equipped with interchangeable 30-, 50-, and 60-kg chest springs and a Corpuls mechanical CC device (GS Elektromedizinische Geräte G. Stemple GmbH, Kaufering, Germany). For each spring resistance, continuous CCs were delivered in two-minute intervals at predefined combinations of CC depth (2.0–6.0 cm) and rate (80–120 compressions per minute). CC quality was quantified using the QCPR score (0%–100%). Across all spring resistances, QCPR scores rose modestly at 3.0–3.5 cm and increased further at 4.0–4.5 cm, where the greatest score variation across preset CC rates and spring resistances was observed. At ≥ 5.0 cm, QCPR scores were consistently high (57%–100%). Overall, CC depth was the primary determinant of the QCPR score, with the highest scores observed at ≥ 5.0 cm. Additionally, we observed QCPR score differences of up to 18% for identical preset CC depth and rate settings with different springs. High QCPR scores were often achieved outside guideline ranges, emphasizing the need to interpret QCPR scores alongside absolute CC parameters and use guideline-compliant metrics as the standard for training and research.

## Introduction

High-quality chest compressions (CCs) play a vital role in improving survival following cardiac arrest. Current guidelines recommend a CC depth of 5–6 cm, a rate of 100–120 CCs per minute, full chest recoil, and minimal interruptions [1, 2]. Accordingly, high-quality CCs are emphasized as a key component of cardiopulmonary resuscitation (CPR) training [3, 4].

Laerdal Medical (Stavanger, Norway) developed the Quality CardioPulmonary Resuscitation (QCPR) scoring system, which enables learners and educators to monitor key CPR parameters and receive immediate performance feedback [5]. QCPR scores are widely used to assess CPR performance in training and resuscitation education research [6–9], with predefined thresholds commonly used to indicate adequate performance [10–14]. However, because the exact mathematical formulation and weighting of the QCPR score are not publicly available [6–9], educators and researchers cannot independently determine how closely the composite QCPR score corresponds to guideline-compliant CCs [1, 2, 15]. According to the manufacturer, the QCPR score for compression-only CPR incorporates several performance metrics, including CC depth and rate, incomplete chest recoil, hand position, number of compressions per cycle and CCs fraction (also referred to as flow fraction). The scoring algorithm uses a non-binary approach, applying progressively greater penalties as performance deviates further from guideline targets [5]. The Resusci Anne QCPR manikin can be fitted with chest springs of different resistance levels to simulate variation in chest wall compliance. However, it is unclear whether or how spring resistance influences the QCPR score [6, 16–18].

We aimed to investigate the association between QCPR scores and preset CC depth and rate across different spring resistances in a manikin model.

## Methods

We conducted an experimental study using a new, previously unused Resusci Anne QCPR manikin (Laerdal Medical, Stavanger, Norway), placed on the floor, and a Corpuls CPR mechanical CC device (GS Elektromedizinische Geräte G. Stemple GmbH, Kaufering, Germany). We conducted two minutes of continuous CCs at prespecified depths and rates. CC depth was increased from 2.0 to 6.0 cm in 0.5-cm increments, and CC rate was increased from 80 to 120 compressions per minute in increments of 5 compressions per minute; the maximum values reflected the upper operational limits of the mechanical CC device. We repeated all attempts using three spring resistances (30, 50, and 60 kg; Laerdal Medical, Stavanger, Norway). The QCPR scoring system was used to evaluate CC quality, with the overall score expressed as a percentage ranging from 0% to 100% [10]. By employing the mechanical CC device, the piston remained in a fixed position over the compression point, thereby standardizing the QCPR hand-position parameter, chest recoil, and CC fraction. Compressions were consistently delivered at each predefined combination of CC depth and rate, minimizing within-condition variability. Data from the Resusci Anne QCPR manikin were collected using the SkillReporter for Tablet application, version 1.4.1 (Laerdal Medical, Stavanger, Norway) installed on a Samsung Galaxy Tab S6 Lite tablet (Samsung Group, Seoul, South Korea).

### Endpoints

We analyzed the agreement between a QCPR score of ≥ 75%, corresponding to the manufacturer-defined threshold for an “Advanced CPR Performer” [19], and guideline-compliant CCs, defined as a depth of 5–6 cm and a rate of 100–120 compressions per minute [1, 2]. Sensitivity analyses were performed using QCPR score thresholds of ≥ 70% and ≥ 80%.

### Statistical Analysis

For all thresholds, sensitivity, specificity, positive predictive value, and Cohen’s kappa with corresponding 95% confidence intervals were calculated. Statistical analyses were performed using the IBM SPSS Statistics graphical user interface (version 29; International Business Machines Corporation, Armonk, NY, USA); therefore, no analysis scripts were generated. Data visualizations were produced using R, version 4.3.1 (R Core Team, Vienna, Austria), and additional figures were prepared using Inkscape, version 1.4.2 (Free Software Foundation, Boston, USA).

## Results

**Fig. 1:**
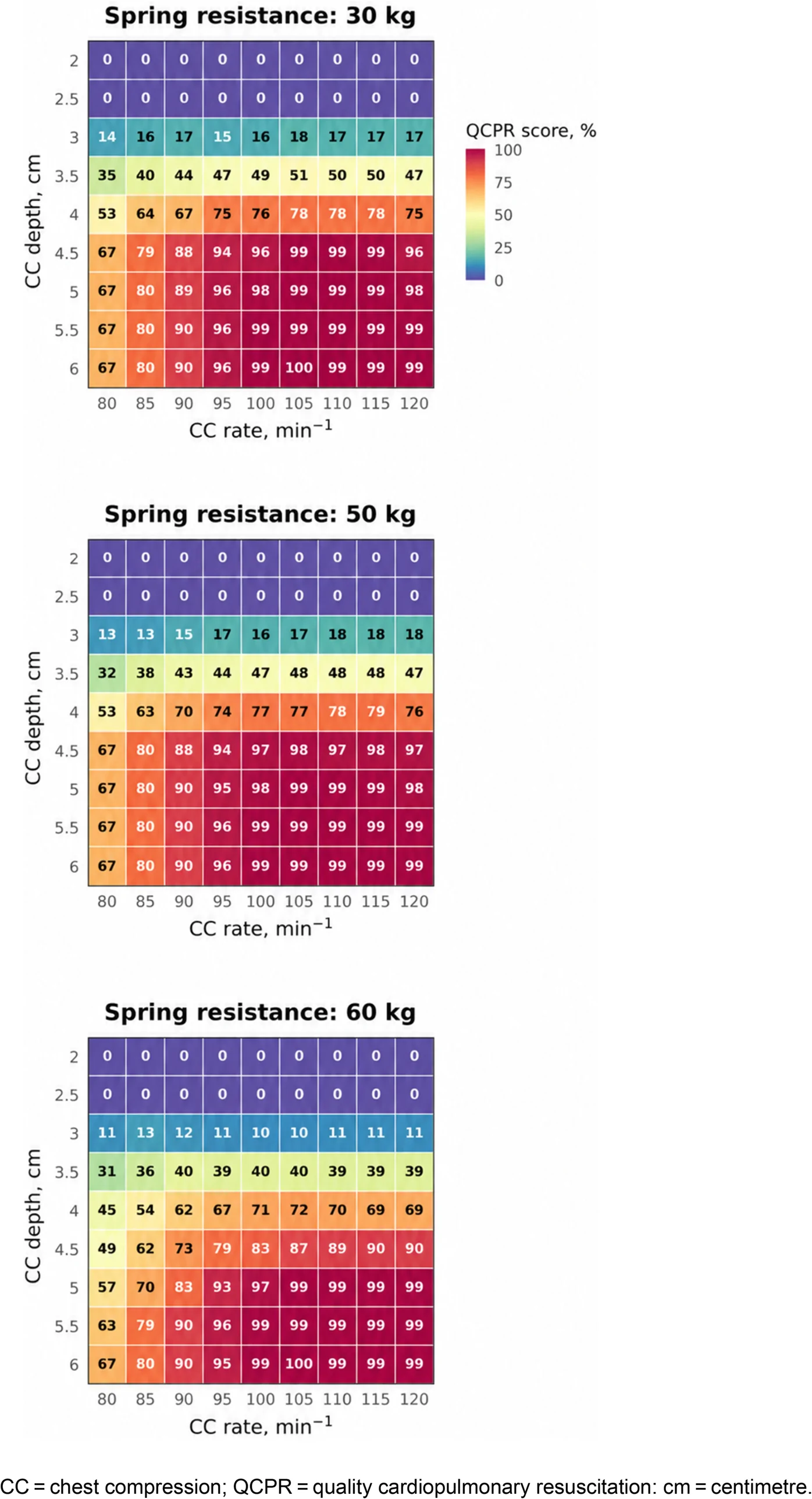
QCPR scores across different chest compression depths and rates using varying spring resistances

We performed a total of 243 individual measurements. At CC depths of 2.0–2.5 cm, QCPR scores were 0% regardless of CC rate and spring resistance. At a depth of 3.0 cm, QCPR scores increased slightly, ranging from 10% to 18% depending on CC rate and spring resistance, with the greatest difference between springs of 8% observed at 105 compressions per minute. At 3.5 cm, QCPR scores improved further, ranging from 31% to 51%, depending on CC rate and spring resistance. A maximum difference of 11% between springs was observed at 105–115 compressions per minute. At a depth of 4.0 cm, QCPR scores increased markedly, ranging from 45% to 79% and showing the combined influence of CC rate and spring resistance, with the greatest difference between springs of 10% observed at 85 and 115 compressions per minute. At 4.5 cm, QCPR scores ranged from 49% to 99%, with most exceeding 80%. Different spring resistances produced QCPR score differences of up to 18% at this depth and at CC rates of 80 and 85 compressions per minute. From 5.0 cm onward, QCPR scores remained consistently high across all conditions, with differences of up to 10% depending on spring resistance. At 5.5 and 6.0 cm, QCPR scores approached the maximum, ranging from 63% to 100% regardless of CC rate or spring resistance; the largest difference between springs was 4% (Fig. 1).

Using 243 CC epochs, 45 (18.5%) were guideline compliant. At a QCPR score threshold of ≥ 75%, sensitivity for identifying guideline-compliant CC epochs was 100%, specificity was 70.2%, positive predictive value was 43.3%, and Cohen’s kappa was 0.47 (95% CI 0.37–0.57), indicating moderate agreement. In sensitivity analyses, a threshold of ≥ 70% yielded a sensitivity of 100%, specificity of 66.7%, positive predictive value of 40.5%, and Cohen’s kappa of 0.43 (95% CI 0.33–0.52). Increasing the threshold to ≥ 80% maintained a sensitivity of 100%, with specificity of 77.3%, positive predictive value of 50.0%, and Cohen’s kappa of 0.56 (95% CI 0.45–0.66).

## Discussion

In summary, CC depth was the primary determinant of the QCPR score. CC rate had secondary effects that were most evident at intermediate depths of 3.5–4.5 cm, whereas its influence became less pronounced once depth reached or exceeded 5.0 cm. Furthermore, we observed QCPR score differences of up to 18% for identical preset CC depth and rate settings when different springs were applied. Agreement between QCPR score thresholds of ≥ 70%, ≥ 75% or ≥ 80% and guideline-compliant CCs was moderate.

Our findings indicate that QCPR scores do not consistently reflect compliance with guideline-recommended CC parameters. At the manufacturer-defined threshold of ≥ 75% for an “Advanced CPR Performer” [19], all guideline-compliant CC epochs were identified; however, specificity was 70.2% and positive predictive value was only 43.3%. Similar findings were observed in sensitivity analyses using thresholds of ≥ 70% and ≥ 80%. Thus, a substantial proportion of epochs meeting the QCPR score thresholds were delivered at CC depths or rates outside the recommended ranges. In an educational setting, this could provide trainees with positive reinforcement despite suboptimal performance and may reduce attention to correcting individual CC parameters. Clinically, such feedback could create an inaccurate perception of readiness to deliver guideline-compliant CPR, although our manikin-based findings cannot be directly extrapolated to patient outcomes.

Previous studies have evaluated CC performance using manikins with different spring resistances, but the association between spring resistance and QCPR scores under standardized device settings remains unclear [6, 16, 17]. In our study, QCPR scores differed by up to 18% across spring resistances despite identical preset CC depth and rate settings. The mechanism underlying this variation remains uncertain. Different spring stiffnesses may have produced subtle differences in the compression waveform, recoil, mechanical oscillation, or sensor response, particularly near nonlinear scoring thresholds. Because the QCPR scoring system is proprietary, the mechanism underlying this variation remains uncertain. Whether the observed differences reflect input from force-sensitive sensors or other effects of spring resistance on compression measurement is unknown. Nevertheless, differences of this magnitude could affect whether performance is classified as meeting a prespecified competency threshold. Ideally, QCPR scores should be independent of manikin spring resistance or adjusted for the selected resistance setting. Until this is established, spring resistance should be reported, and QCPR scores should be interpreted alongside absolute CC parameters.

Future iterations of QCPR feedback systems could account for the observed influence of manikin chest compliance. Ideally, identical compression performance should produce comparable feedback across different spring-resistance settings. However, rather than introducing further adjustments to an opaque composite score, future systems could prioritize transparent, guideline-based reporting. This approach, consistent with the Resuscitation Education Utstein recommendations [15], may improve comparability across manikin configurations and training environments while allowing users to identify the specific reasons for suboptimal performance.

High-quality CCs are the cornerstone of improving survival following cardiac arrest, and current guidelines emphasize their delivery with an adequate depth of 5–6 cm, a rate of 100–120 CCs per minute, full chest recoil, and minimal interruptions [1, 2]. While guidelines remain the primary reference, studies correlating CC depth and rate with patient survival provide complementary insights [20]. In the manikin, QCPR scores increased steadily with depth and plateaued at 5.0 cm and above, becoming nearly independent of rate. Importantly, we were unable to detect depths >6 cm why we cannot determine whether the QCPR scoring system penalizes excessive CC depth. If no such penalty is applied, the score could potentially fail to distinguish guideline-compliant CCs from excessively deep CCs associated with an increased risk of injury. In contrast, a previous study showed the highest survival at CC depths of 4.5–5.0 cm and CC rates of 100–110 per minute, with outcomes declining with both shallower and deeper CCs and with slower and faster rates [20]. These findings suggest that manikin feedback systems may overemphasize deeper CCs and broader rate tolerances compared with the narrower “sweet spot” observed in clinical practice and as recommended in resuscitation guidelines.

Current QCPR scoring system rely on closed algorithms, which limit transparency and restrict adaptation to new evidence. Some researchers have proposed open-access, AI-driven scoring models trained on large real-world datasets [21–23]. However, uniform guideline-compliant reporting should remain the foundation for education and research, as it facilitates comparability across studies and training programs. Innovations in algorithm design may complement these standards but should not replace guideline-based metrics.

Accordingly, CPR performance could primarily be assessed and reported using transparent, guideline-based measures, including the percentages of compressions with guideline-compliant depth, guideline-compliant rate, complete chest recoil, and overall guideline compliance. The composite QCPR score, if reported, should be treated only as supplementary information and should not be used alone to determine competence [15].

### Limitations

Although our experiment involved 243 individual measurements, each lasting two minutes, we performed only one two-minute CC interval for each depth, rate, and spring-resistance setting. However, using a mechanical CC device reduced variability. CC depth and rate represented the preset settings of the mechanical compression device and were not independently recorded by the QCPR scoring system. Although identical settings were applied across spring resistances, small differences in the actual delivered CC depth, rate, or waveform cannot be excluded. Consequently, the observed score variation indicates an association with spring resistance under standardized device settings but does not establish a direct effect of spring resistance on the QCPR scoring system. We did not independently measure CC force or the complete CC waveform and therefore cannot determine whether score differences arose from CC dynamics, sensor behavior, or the proprietary scoring algorithm. We studied only continuous CCs and therefore cannot infer QCPR scores for CPR involving rescue breaths. The maximum tested depth was limited to 6.0 cm by the operational range of the mechanical CC device; therefore, we could not determine whether the QCPR scoring system penalizes excessive CC depths beyond the guideline-recommended range. All measurements were performed using a single manikin; therefore, unit-specific calibration or sensor behavior cannot be excluded. However, the manikin was new and previously unused, and it received less than 10% of the approximately 500,000 CCs after which the manufacturer recommends replacement of relevant components [24]. No visible damage, malfunction, or interruption in QCPR data collection was observed. Nevertheless, progressive mechanical or sensor effects cannot be completely excluded, and studies using multiple manikins are needed to confirm the reproducibility of our findings.

## Conclusion

QCPR scores were primarily associated with preset CC depth, with smaller variations observed across preset rates and spring resistances. High scores were often achieved outside guideline-recommended ranges. Therefore, QCPR scores should not be reported or interpreted in isolation but could be accompanied by absolute CC depth and rate values, with guideline-compliant parameters remaining the primary standard for training and research. 

## Data Availability

The complete dataset underlying this study and the R code reproducing the reported statistical analyses are publicly available in Zenodo at https://doi.org/10.5281/zenodo.22749343 .

## References

[1] Berg KM, Bray JE, Djärv T, Drennan IR, Greif R, Liley HG, et al. Executive Summary: 2025 International Liaison Committee on Resuscitation Consensus on Science With Treatment Recommendations. Circulation 2025;152:S2–22. 10.1161/CIR.0000000000001361. 41122844

[2] Greif R, Abelairas-Gómez C, Böttiger BW. European Resuscitation Council Guidelines 2025: Executive Summary. Resuscitation 2025;215. 10.1016/j.resuscitation.2025.110770. 41117573

[3] Nabecker S, Raad T, Abelairas-Gómez C. European Resuscitation Council Guidelines 2025: Education for Resuscitation. Resuscitation 2025;215. 10.1016/j.resuscitation.2025.110739. 41117567

[4] Nallamothu BK, Greif R, Anderson T, Atiq H, Couto TB, Considine J, et al. Ten Steps Toward Improving In-Hospital Cardiac Arrest Quality of Care and Outcomes. Circ Cardiovasc Qual Outcomes 2023;16:e010491. 10.1161/CIRCOUTCOMES.123.010491. PMC10659256 37947100

[5] Laerdal Medical. CPR scoring explained 2015.

[6] Cortegiani A, Russotto V, Montalto F, Iozzo P, Meschis R, Pugliesi M, et al. Use of a Real-Time Training Software (Laerdal QCPR^®^) Compared to Instructor-Based Feedback for High-Quality Chest Compressions Acquisition in Secondary School Students: A Randomized Trial. PLOS ONE 2017;12:e0169591. 10.1371/journal.pone.0169591. PMC5215847 28056076

[7] Labuschagne MJ, Arbee A, de Klerk C, de Vries E, de Waal T, Jhetam T, et al. A comparison of the effectiveness of QCPR and conventional CPR training in final-year medical students at a South African university. Afr J Emerg Med 2022;12:106–11. 10.1016/j.afjem.2022.02.001. PMC8885445 35251921

[8] Huang W-H. CPR Skill Achievement in Police First Responders: A Cross-Sectional Study Using QCPR and Kirkpatrick’s Model. Disaster Med Public Health Prep 2025;19:e222. 10.1017/dmp.2025.10164. 40765111

[9] Barbe O, Hervieu L, Lefrançois E, Sénémeaud C, Michel F, Macrez R, et al. Effect of in situ coaching on cardiopulmonary resuscitation quality: a multicenter randomized simulation study. Resusc Plus 2026;29:101352. 10.1016/j.resplu.2026.101352. PMC13199809 42199518

[10] Nicolau A, Jorge I, Vieira-Marques P, Sa-Couto C. Influence of Training With Corrective Feedback Devices on Cardiopulmonary Resuscitation Skills Acquisition and Retention: Systematic Review and Meta-Analysis. JMIR Med Educ 2024;10:e59720. 10.2196/59720. PMC11695954 39699935

[11] Pallas J, Miller M, Hicks S, Newton P, Chu G, Smiles JP, et al. Balancing perfectionism with expansion in community CPR training. Resusc Plus 2026;29:101301. 10.1016/j.resplu.2026.101301. PMC13081160 41993773

[12] Pallas J, Miller M, Hicks S, Newton P, Chu G, Smiles JP, et al. Exploration of ‘generational’ peer-led CPR training in the Australian community using blended learning approaches: a pilot randomised controlled trial. Resusc Plus 2026;27:101190. 10.1016/j.resplu.2025.101190. PMC12828363 41583924

[13] Oermann MH, VanRiel YM, Wagner RW, Whittington KD, Baker M, Stieve DE, et al. Maintenance of CPR skills among nursing students trained using Resuscitation Quality Improvement^®^ program. Resusc Plus 2024;20:100782. 10.1016/j.resplu.2024.100782. PMC11426146 39328898

[14] Dudzik LR, Heard DG, Griffin RE, Vercellino M, Hunt A, Cates A, et al. Implementation of a Low-Dose, High-Frequency Cardiac Resuscitation Quality Improvement Program in a Community Hospital. Jt Comm J Qual Patient Saf 2019;45:789–97. 10.1016/j.jcjq.2019.08.010. 31630977

[15] Cheng A, Lauridsen K, Greif R, Finn J, Eastwood K, Yeung J, et al. Uniform reporting of outcomes from resuscitation education research: the resuscitation education Utstein style: a consensus report from the International Liaison Committee on Resuscitation. Resuscitation 2026;223. 10.1016/j.resuscitation.2026.111047. 42009583

[16] Kao C-L, Tsou J-Y, Hong M-Y, Chang C-J, Su F-C, Chi C-H. Effect of thoracic stiffness on chest compression performance - A prospective randomized crossover observational manikin study. Heliyon 2022;8. 10.1016/j.heliyon.2022.e10990. PMC9573907 36262289

[17] Thurlow K, Rehnberg L, Ivetić J, Russomano T, Levic S. Elbow flexion enables rescuers with low BMI to deliver chest compressions in compliance with CPR guideline recommendations. Sci Rep 2026;16:9723. 10.1038/s41598-026-39671-5. PMC13013621 41708689

[18] Weidenauer D, Hamp T, Schriefl C, Holaubek C, Gattinger M, Krammel M, et al. The impact of cardiopulmonary resuscitation (CPR) manikin chest stiffness on motivation and CPR performance measures in children undergoing CPR training—A prospective, randomized, single-blind, controlled trial. PLOS ONE 2018;13:e0202430. 10.1371/journal.pone.0202430. PMC6095555 30114240

[19] Support for SimPad PLUS with SkillReporter. Laerdal Med n.d. https://www.laerdal.com/products/simulation-training/resuscitation-training/simpad-plus-with-skillreporter/support (accessed September 15, 2026).

[20] Duval S, Pepe PE, Aufderheide TP, Goodloe JM, Debaty G, Labarère J, et al. Optimal Combination of Compression Rate and Depth During Cardiopulmonary Resuscitation for Functionally Favorable Survival. JAMA Cardiol 2019;4:900–8. 10.1001/jamacardio.2019.2717. PMC6694399 31411632

[21] Ko S, Lee Y, Choi M, Choi D, Lee CA, Hou J-U. Harnessing optical flow in deep learning framework for cardiopulmonary resuscitation training. Expert Syst Appl 2024;238:121775. 10.1016/j.eswa.2023.121775.

[22] Ecker H, Adams N-B, Schmitz M, Wetsch WA. Feasibility of real-time compression frequency and compression depth assessment in CPR using a “machine-learning” artificial intelligence tool. Resusc Plus 2024;20:100825. 10.1016/j.resplu.2024.100825. PMC11570746 39559731

[23] Chotikunnan P, Imura P, Thongpance N, Chotikunnan R, Suriwong T, Phantong T, et al. Designing and Building an Interactive CPR Training Manikin Accessory Kit. Int J Eng Appl IREA 2025;13:341–9. 10.15866/irea.v13i4.24894.

[24] Resusci Anne QCPR Periodic Maintenance Kit. Laerdal Med n.d. https://laerdal.com/gb/item/171-20050/ (accessed September 7, 2026).

